# Cases from Practice

**Published:** 1883-05

**Authors:** R. G. Bogue

**Affiliations:** Chicago


### ARTICLE III.

### CASES FROM PRACTICE, BY R. G. BOGUE, M. D., CHICAGO.

*Fibroid in Abdominal Wall.*

Mr. Allen, age 28, about two and a half years ago noticed a small lump in the abdominal wall, a little above, and to the left, of the navel. It steadily increased in size, but was neither tender nor painful, the only discomfort experienced coming from the hard bunch being pressed upon by the clothing and hit by anything held against that part of the abdomen. Of late it had increased noticably in size, and being alarmed about its growth, he sought advice. Upon examination, a tumor about the size of a fist could be felt evidently in the abdominal wall, occupying a place a little above and to the left of navel, extending to near the border of the ribs, not movable laterally, nor upward nor downward; could be pressed backward somewhat, but not lifted forward; the skin freely movable over it, resonance behind it, no tenderness, and not painful. Diagnosis, probably a fibroid growing in the sheath of the rectus, or in the hard part of the abdominal wall. Extirpation was advised.

September 23d, assisted by Doctors John Bartlett and G. W. Reynolds, I removed the tumor under the carbolic spray, the patient being etherized, after dividing the skin and found the tendon in front of the muscles very much thinned. It covered the tumor and was adherent to it, except at the outer border, where it seemed to have been separated and crowded downward. The muscular fiber was exceedingly thin, and but little was seen except along the border of the ribs and at the lower edge of the tumor where the rectus seemed adherent to it. The tumor was separated from its attachments and enucleated from its bed and removed without difficulty. When the tumor was removed there was left a cavity with tendinous borders, mostly, and which had for a floor the peritoneum. Two or three vessels were tied with catgut, and the wound closed by passing through all of the tissues to the peritoneum, sutures of silk-worm gut, thereby drawing the tendinous borders of the opening as near together as possible. A drainage tube was left in the lower angle of the wound, and a

Lister gauze dressing applied. The dressings were changed at the end of every twenty-four hours; they were soiled somewhat by a moderate discharge of bloody serum; the drainage tube was removed. The whole wound healed by first intention, there being no soiling of the dressings except the bloody serum of the first day. The sutures were removed at the end of a week, having held the parts very nicely, and with very little irritation and no suppuration around them. The patient suffered during the first forty-eight hours with quite severe spells of colic, caused by gas within the intestines. This was quieted by morphine and a mild laxative. There was no other annoyance, and in ten days he was able to be out of bed. He is to wear for a prolonged time a snugly fitting band to protect the weakened part of the abdominal wall.

I would call attention to the silk-worm gut for sutures. It is more easily or readily used for closing a wound than silver wire; is a source of as little irritation and may be left in place for any necessary length of time, up to two weeks, provoking no suppuration, and is readily removed, more so than wire. Either of them is, we think, far preferable to silk. The drainage tube has served its purpose; allowing the escape of blood and serum directly after operation, by the end of 24 to 48 hours, and should then be removed, in most cases.

*Excision of Knee-Joint for Chronic Adhesive Inflammation—Recovery.*

Case I. Fannie B., aged 16 years, came under my care March 22, 1877. Four years previously she had experienced sudden pain and stiffness in the right knee, which soon became hot and swollen. At the end of three months, having meanwhile made cold applications, she had regained fair use of the limb. For two years she suffered no inconvenience, but, after convalescence from typhoid fever, she was compelled to use crutches, the knee being stiff, swollen, tender and painful; was well nourished, and general health good. The excision of the knee was determined upon. The usual horse-shoe flap was made, when it was found that there was no ulceration, but that the synovial cavity was nearly obliterated by bands of adhesion; the inner condyle was enlarged by chronic inflammation. In consequence of this enlargement the patella was apparently displaced outwards upon the external condyle. The articular surface of the tibia, the patella and the condyles of the femur were removed; the flap was replaced; several sutures were inserted; the limb was dressed on a long posterior splint, which was suspended from a frame; a drainage-tube was inserted into the angle of the wound, which was covered with a carbolized compress and oiled silk. Anodynes were administered as needed.

During the two days following the patient had severe nausea and vomiting, but after that time presented not a single unfavorable symptom. Her pulse ranged from 90 to 116; her temperature from 100 to 102.

April 18.—Hodgen's anterior splint was substituted for the straight splint. On the 20th this was replaced by a plaster of Paris dressing, which was made to grasp firmly the thigh and the leg, and was especially strong behind the knee, but was cut away above so as to leave the wound exposed.

June 4.—The drainage-tube was removed.

June 14.—Patient began to walk with the aid of crutches.

July 4.—A small piece of bone was removed from one of the sinuses still open.

Several small sinuses remained obstinately open until the middle of October. During her long sojourn in the hospital patient's general health continued excellent, and, upon her departure, November 2d, she walked readily with a single cane.

Four years later the patient was seen. The leg had given no trouble and was a good and useful limb, but short about four inches.

*Excision of Hip-Joint—Recovery.*

Willie J., 10 years old, was admitted March 12, 1877. Three years previously he had fallen down and hurt his hip, after which time he was unable to rest the entire plantar surface of the foot on the ground, but transmitted his weight through the toe. A year later there was a discharge of pus from an opening at the posterior border of the great trochanter. The sinus closed and re-opened several times.

On admission the right leg and thigh were found somewhat atrophied ; the thigh slightly flexed and adducted ; the hip-joint anchylosed. There was a sinus at the posterior border of the great trochanter and a large fluctuating tumor at the upper and anterior aspect of the thigh. The patient complained of great pain in the right knee.

Examination under an anæsthetic detected no carious bone the sinus leading down simply to the capsular ligament.

April 4.—An incision was made over the great trochanter and the upper extremity of the femur removed just below the lesser trochanter. The head and neck were mostly ulcerated away, a mere shell of bone remaining. Alcohol dressings were applied; the limb was extended by weight and pulley, counter extension being made by elevating the foot of the bed.

During the next week the pulse ranged from 120 to 140; the temperature from 101° to 104°. The general condition gradually improved, the pulse and temperature fell, the discharge continued profuse and on April 18, a drainage-tube was inserted. April 24, an abscess was evacuated below Poupart's ligament. June 6 to 26 he complained of more or less pain in the right knee.

July 7.—Extension was removed in consequence of ulceration beneath the straps, but was renewed August 10. September 1, patient was allowed to leave his bed and to walk around on crutches. November 6, several fragments of carious bone were removed, the discharge still continuing. December 15, a section of the complete circumference of the femur (the cut end) one-half inch long escaped from a sinus on the outer side of the hip.

There still remains (February 8) two sinuses leading down to carious bone. The boy enjoys good health and exhibits great activity upon two crutches.

*Excision of Knee-Joint for Chronic Destructive Inflammation—Pyæmia—Death.*

Peter J., 26 years old, was admitted Nov. 2, 1877. Five years previously he had been thrown from a horse, sustaining an apparently slight bruise of the left knee. With the exception of a little soreness in walking for two or three days, he experienced no inconvenience. Three years later, without any provocation, so far as he knows, the knee became red, swollen and tender, and after some days there burst from the knee, just below the outer border of the patella, a little yellow, watery liquid. There was, for two or three months following, a slight but constant discharge of similar liquid, at the end of which time the sinus closed.

Three months prior to admission, without the infliction of any violence, the knee became so painful that he was compelled to keep it quiet, and in this condition he entered the hospital, Hot fomentations were applied with the effect of still further reducing the swelling.

Nov. 27.—Patient was etherized and an exploratory incision was made into the joint revealing about two ounces of pus and extensive ulceration of the articular cartilages and bone surfaces. The usual semicircular flap was then raised and the condyles of the feurmr, patella and the articular surface of the tibia were at once removed. The flap was replaced; sutures inserted; the limb was placed upon a long posterior splint, and alcohol dressings were applied.

The patient had no untoward symptom until the morning of Dec. 3, when there occurred a slight chill. A second, more violent, occurred Dec. 5. His pulse and temperature remained high, and in a few days frequently recurring chills, profuse perspiration, a pinched countenance, cessation of suppuration rendered unmistakable the toxæmic condition. Death occurred from pyæmia Dec. 20.

The cut surface of the tibia was found to be granulating, that of the femur sloughing.

*Abscess in Lower End of Tibia.*

Annie Martin, aged 29, when about nine years of age fell, bruising the right shin. It gave her but little pain at first, but in the course of three or four weeks an abscess had formed over the front of the middle part of the tibia, which remained open for two or three years. Occasionally small bits of bone came from it.

At the end of this time it closed, and remained so for about twelve or thirteen years. A small abscess formed seven years ago which soon closed; and about five years ago another formed at seat of the former ones, which was open for several months. Three years ago ago an abscess formed near the upper end of the tibia, which soon closed after opening. About one year ago the lower end of the tibia began to enlarge and became painful. When I first saw it there were numerous cicatrices along the front of the tibia from the old inflammation, of superficial necroses which had occurred. These cicatrices were adherent to the bone, but there was no tenderness of the bone except at the lower extremity, which was enlarged, moderately tender upon pressure and the seat of severe pain, especially at night; no particular redness of the skin. It had been gradually enlarging, becoming more and more uncomfortable, and, finally, painful for about a year. From the even and uniform enlargement, absence of soreness of the skin, although the bone coverings were sensitive and the deeply seated and severity of the pain, it was thought there must be an abscess in the lower end of the tibia.

Up to this time, the latter part of August, 1879, she had continued at her work as chamber-maid and table-waiter in a hotel. It was treated for about a week by rest in an elevated position enveloped in a warm anodyne fomentation. No relief being obtained from this treatment, it was decided to make an exploration of the bone.

Sept. 4, 1879.—Patient etherized; an incision made through the coverings of the bone over the most prominent part of the swelling, just above and a little to the front of the middle of the base of the internal maleolus. The periosteum was found thickened. The bone was pierced with a Brainard drill, and, after boring to quite a depth, a cavity was reached. On removal of the drill a drop or two of pus welled up through the opening, which established the fact of an abscess. With a trephine a button of bone was removed, uncovering a cavity which would hold about half an ounce of fluid, about which quantity of pus was removed. No bone fragments were found. The cavity was cleansed, a tent left in and ankle enveloped with an antiseptic wet dressing. After a few days a wax plug was fitted into the wound and covered with oakum. . It was cleansed daily. There was very little pain after the operation. Oct. 2, a point of bone was noticed in the granulations and, on removal, proved to be a ring of bone which had necrosed from about the opening made by the trephine.

The cavity gradually filled with granulations; the wound cicatrized, and by Nov. 13, when the patient was discharged, it had healed; and now, at the expiration of a year, the limb is entirely useful, not painful and not tender. There is enlargement remaining.

